# The Management of Safety Situations and Assumable Risk during Transitions and Recesses in Different Types of Schools

**DOI:** 10.3390/ijerph19074117

**Published:** 2022-03-30

**Authors:** Òscar Flores, Anabel Ramos-Pla, Isabel del Arco

**Affiliations:** Department of Pedagogy, Faculty of Education, Psychology and Social Work, University of Lleida, 25001 Lleida, Spain; oscar.flores@udl.cat (Ò.F.); isabel.delarco@udl.cat (I.d.A.)

**Keywords:** school recess, transition, safety, risk, education

## Abstract

The present study aims to analyze the situations of safety and risk perceived during the school transitions and recesses at different types of schools and to determine the level of empowerment of the students to manage situations of risk. The novelty of the study lies in analyzing the best strategy for dealing with risk situations during school transitions and recess. For this purpose, different situations in different types of schools are analyzed. An observational methodology was utilized. The sample was composed of a total of 23 schools, with 69 different observations conducted at different times of the day (morning recess and noon recess, on sunny days and rainy days). The results show that the morning recesses were the safest, and that the schools that serve a great number of at-risk students offered the greatest safety during recess and its transitions. With respect to the level of empowerment of the students, it was observed that they managed the possible risks when the safety guidelines were clear. The study provides evidence showing that when students are clearly aware of recess rules and guidelines, the management of risk situations improves.

## 1. Introduction

This article is based on the idea already expressed by [1,2], who propose the need for schools to promote safe work and, at the same time, raise awareness of the main risks to help avoid them. To this end, it is necessary to focus on students, making them aware of and involved in the management and prevention of risk factors, and, at the same time, to involve teachers in the need to consider health and safety as teaching elements to generate preventive behaviors that result in the promotion of health.

The authors have considered it to be important to study the concrete reality and see how these issues are developed in schools, specifically during recess. We believe that making a diagnosis of how safety issues are being addressed in different school realities helps to provide evidence that can be used to guide the planning of safe school transitions and breaks, aimed at promoting coexistence and the physical, social, and emotional development of students.

To carry out this analysis of the playground environment with a specific focus on safety, resources, pupil participation, adult participation, and pro-social/anti-social behavior and pupil empowerment, a valid and reliable assessment tool is used, the GRF-OT scale, which has been piloted in other international educational contexts. This scale helps us to make diagnoses in order to guide further interventions.

### 1.1. Recess at School

Recess at school takes place during the school day to provide the children with a change from academic activities. In the field of education, rest times within the academic activities at school are necessary for the healthy development of children and as tools to promote learning [3,4,5,6], bringing health, social, cognitive, and developmental benefits.

The regulations indicate that recess is part of the school day, so that it should be as important and have the same value as other areas of the curriculum [7]. It is a space and a period of time in which the students can rest from the academic sessions to play and socialize with their peers, allowing them to gain energy to continue with the classroom sessions that take place after recess [8,9]. Nevertheless, in our context, research studies indicate that, in general, recess at school is not considered to be a first-order educational space [10,11].

The activities conducted at recess can be open or designed by the teachers, especially to ensure participation [12]. A good organization of play time can help improve psychomotricity and physical activity, and can also have an effect on the social, cognitive, and emotional areas of the students [13,14,15].

It is mandatory that the school yard space used for recess comply with technical requisites to guarantee the safety of the children, and schools must manage this safety with contextual and organizational criteria [16].

This study provides new information about which of the different types of recess that take place at school present the best levels of safety, comparing different types of schools. It also sheds light on the levels of student empowerment and its influence on managing risk situations during school recess.

### 1.2. Safety and Risks in School Recess

Traditionally, school recess is the period of time during a school day with the greatest number of risks for children, because it occurs in an open and free space, created for playing, in which students can perform motor actions that are very different from those that are performed in the classroom [17]. In fact, some studies have shown that the space utilized for recess at school is one of the main places where injuries occur [18,19].

In this paper, when we talk about the concept of “risk”, we do not limit ourselves to the possibility or danger of the child suffering a physical injury (due to equipment, in the space itself, through interaction with peers, etc.), but we align ourselves with authors such as [20,21,22], understanding risk to be a situation in which children can recognize and evaluate a challenge and decide the course of their action. Therefore, we place ourselves in the range of risk perception and risk management. We also relate this risk to conflict management, i.e., to problems arising from the interaction between boys and girls (arguments, fights, etc.), and to the management that can be performed by schools. The design of the space where recess takes place, the maintenance, the availability of equipment, and the participation of adults contribute to the avoidance of safety problems and exclusion practices [23,24]. Likewise, the intervention or not of teachers directly affects the behavioral patterns of children during recess [25,26,27].

Another important element is the transition, that is, the moving from the classroom to recess, or the entry and exit from the school premises. Traditionally, the students form a line at the start and end of different activities: to enter, to exit, to go to recess, to return to the classroom, to go to the sport courts, to obtain food, or to go to the library [28]. Ultimately, schools use mechanisms of order that indicate the start and end of different activities, and which allow them to maintain a certain level of student safety.

Different factors have an influence on the safety of the school recreational spaces [29]: personal influences (taking risks, preventing boredom, and bad behavior); interpersonal relations (responsibilities of the teacher, teacher support, support between students, and bullying problems); influences of the physical space (surfaces, protection against adverse climatic conditions, safe structures, and protection equipment); and political and organizational influences (designated play areas, guidelines, level of supervision, and maintenance). Therefore, in the area of safety at recess, various agents are responsible for looking after the physical and moral integrity of the children.

Research studies have shown that although it is probable that accidents may occur at school, most of them could be avoided. In general, the accidents during recess occur because the fixed or mobile equipment (climbing ropes, support bars, etc.) do not meet the safety guidelines or the space is not well-designed [30,31].

Nevertheless, the traditional concept of school safety has been redefined and improved because, to protect users and guarantee healthy environments, it is necessary to consider multiple dimensions that include different types of risks: static risks (the conservation and maintenance of the installations by groundkeepers, etc.) and dynamic risks (those risks that are associated with the process of interaction with people) [32,33].

The evolution of the concept of risk has also brought with it the publication of works that are opposed to an approach that is free of risks for children’s play, focusing the perspective into an approach of cost and benefit analysis [34]. Zero risk does not exist, so we must advocate for the empowerment of children to facilitate its management. Favoring participation, informing, preventing, and awareness should be basic aspects for improving safety at school recesses [35,36,37,38,39].

### 1.3. School Recess Guidelines

In Spain, Royal Decree 132/2010 [34] dictates the minimum requirements that must be met by the common use facilities at primary and secondary schools. Article 3 establishes that the school playgrounds must have a surface area of at least 900 square meters, be partially covered and, if needed, be able to be used as a sports field. It also establishes the safety requirements of the equipment and facilities at schools (the size of the playground is calculated considering the number of classes), as well as the recess periods for the children. The Spanish guidelines are based on a European guideline [40], which specifies the general safety requirements for the equipment and surfaces of children’s public parks that are permanently installed.

Within the context of Catalonia, the recess activities are regulated through different regulations [41,42,43,44]. Recess is described as an educational activity that is part of the school schedule, which must respect the principles of the education project of the center. The outside playground must have a minimum of 2 square meters per child for simultaneous use, and a minimum of 75 square meters.

As for the creation of new school buildings, the Department of Education of Catalonia establishes regulations such as the inclusion of water fountains and benches in the playgrounds and access to bathrooms outside of the main school building [44]. Additionally, other guidelines establish that the entry to the playgrounds must be paved and that access should be easy, especially if the entrances must be used by very young children [45].

Ultimately, although the guidelines are clear and well defined, questions still remain that must be answered by research studies, such as: In terms of different school typologies, what are the situations of safety or risk that arise in school transitions and recess? Do pupils show the autonomy and empowerment necessary to manage these situations? It is within this context that we find the significance of the present research contribution, with respect to comparing and contrasting the levels of perception of safety and the tolerance of risk in different types of school, as well as the student’s management of different situations or risks.

## 2. Methodological Design

### 2.1. Objectives

To ensure the traceability of the study, we start from these objectives:

- To analyze the situations of safety and risk perceived during the school transitions and recess according to the type of school.
- To determine the empowerment of the students for managing situations of risk.

### 2.2. Methodological Approach

In this study, an observational method was utilized, with the application of the Great Recess Framework (GRF-OT) scale [45,46,47]. This scale is a valid and reliable evaluation tool, created for measuring factors associated with recess and the behaviors of its protagonists in this context. Its authors describe it as a tool that allows educators and researchers to evaluate and plan the school recess with a holistic approach.

Before its use, the Great Recess Framework (GRF-OT) scale was first translated from English into Catalan and then back-translated into the original language of the scale, i.e., from Catalan into English. The back-translation was carried out by a different translator who is a native speaker. This was performed in order to check the accuracy, precision, and equivalence of the translation by comparing both versions. In this way the GRF-OT.Cat version is equivalent to and conforms to the original version. The translated version was subjected to content validity analysis by 10 judges, who evaluated the uniqueness, suitability, and degree of importance of each item. Additionally, considering that there would be two observers collecting the data, inter-rater reliability (inter-rater reliability) was calculated for each item, for each dimension, and for the overall scale. The significance of the difference between the two was tested and no significant differences were found (all *p*-values > 0.650). Therefore, we can report an absolute percentage of agreement, obtaining for each item, for each dimension, and for the scale as a whole an inter-rater reliability between 0.870 and 0.951. The final scale, GRF-OT.Cat, was thus created and used in the present study.

This final version is composed of 17 items, which are scored with a Likert scale of 4 points, where 1 represents a bad situation/state of the education center, and 4 a good situation/state. These 17 items were configured around 5 dimensions:

- D1. Safety and structure: 5 items.
- D2. Adult engagement and supervision: 4 items.
- D3. Student behaviors: 5 items.
- D4. Transitions: 2 items.
- D5. Physical activity: 1 item.

In order to achieve the proposed objectives, certain dimensions and items were selected from the entire GRF-OT scale. From D1, the items related to structures, the delimitation of spaces, and resources were selected. D2 was not considered. From D3, items related to the rules of the game and strategies for resolving conflicts were selected. D4 was considered in its entirety. D5 was not considered. Table 1 shows the dimensions and items that were used.

The second step consisted of the training of two observers to make them experts on data collection, thus ensuring the objectivity of the information provided, as the same two observers collected the data.

The third and last step was the establishment of key application criteria of the GRF-OT.Cat scale, with the consent of the participating schools. Thus, considering that the original version of each dimension had a greater degree of stability when observations were made on different days (minimum 3 days) and at different times throughout the school day, different moments in time were established. The observations were made at three different days and times during the split morning/afternoon school day: two observations at recess during the morning schedule, corresponding to days with different weather (first observation = sunny day, second observation = rainy day), and an observation during the noon recess (third observation = noon).

The informed consent from the school management and teaching staff was obtained to access the school. They approved the specific access days of the observers.

### 2.3. Information Collection Strategy: Characterization of the Sample

This study was contextualized in Catalonian schools. Through probabilistic stratified sampling, a total of 23 Catalan schools were selected. These were of different types, according to the classification from the education administration. The resulting sample was composed of:

- Six high-complexity public schools, which serviced socio-economically vulnerable populations, and which did not have satisfactory results in basic competency tests.
- Six ordinary public schools, which serviced a population that could be classified within a medium or medium–high range from the socio-economic point of view, and which obtained satisfactory or very satisfactory scores in the basic competency tests.
- Six subsidized schools (public–private agreement), with characteristics similar to ordinary schools, and which only differed in the type of center ownership.
- Five RAS schools (rural area schools), incomplete rural schools which serviced a rural population with a medium socio-economic level, and with satisfactory or very satisfactory scores in the basic competency tests.

The differences in the type of school allowed us to establish correlations, considering the education center’s own characteristics. The sampling procedure was as follows:

1. To identify the population. In our case, the population consisted of pre-primary and primary schools in Catalonia.
2. Construction of clusters according to the socio-economic–cultural characteristics and results in the assessment of basic competences of the different schools.
3. Sample selection. From each cluster, a convenience sample was taken to select a number of schools. The sampling was adjusted according to the availability of the selected schools in each cluster and to ensure that all schools had playgrounds adapted to the standards.

In Catalonia, all schools (public and subsidized) must comply with the safety and risk prevention regulations established by the administration. The most notable differences are in the type of pupils they take in.

### 2.4. Process and Data Analysis

A descriptive analysis of the data was performed. With all items in Likert format (or a similar ordinal scale), the general descriptive analysis was performed with: frequency count, mean, median, and standard deviation. The possible adjustment to statistical normality was contrasted with the Kolmogorov–Smirnov test. In the presence of marked asymmetries, the centrality of the variables is presented in these results together with the values of the means and medians. The lack of statistical normality determined the use of nonparametric tests in the inferential analyses performed.

The Friedman test (repeated measures) was used with the 3 observations to verify the absence of statistical significance between them and to justify the calculation of a total mean value for each item.

For the comparative analysis of the average values (mean/median) of the variables of the dimensions according to school typology (groups independent of each other), the Kruskal–Wallis H-test was used.

In all these tests, the significance level set was the usual 5% (significant if *p* < 0.050).

## 3. Results

The results are presented in two sections. On the one hand, we analyzed the data referring to dimensions D1 and D4, which respond to the first objective of the study, and on the other hand, the results refer to dimension D3, which responds to the second objective of the study.

It is important to remember that we are interested in knowing if there are points of insecurity that can generate risks (both in spaces and with the resources destined for school breaks), as well as in transitions. It is important to analyze whether different situations and responses are generated depending on the type of school.

In a second section, data on student empowerment in managing possible risk situations are presented.

### 3.1. Safety and Risks Perceived during the Transitions and Recesses

The different observations were conducted using the Likert scale for each item, expressing the results as a percentage (*n* = 23), and the differences for each type of school. In D1: Safety and structure, it is observed that in all the items, most of the observations are concentrated in the values of 3 and 4, the highest ones. This underlines that in a global manner, no safety problems were found relating to the issues evaluated in these items.

The comparison of these items between the three observations performed (Table 2) showed that no differences were found that were statistically significant (with a *p* > 0.05) in the first three items.

For item 1.3 (Safety of fixed and non-fixed recess equipment), a statistical significance was found (*p* < 0.05), which was due to the mean from the first observation being slightly higher (3.78) than in the other two observations (3.52), despite the medians being equal (4 points). The estimated effect size for this difference was 17.4% (which is considered to be large). Thus, it seems that during the morning recess, the equipment and resources provided (fixed and non-fixed equipment) were used correctly.

When considering the types of schools studied (Table 3) with the results from the comparison test, some significant results (at least *p* < 0.05) were found, along with other variables that were found to be not significant (*p* > 0.05), although they were close (*p* < 0.10).

**Figure 1:**
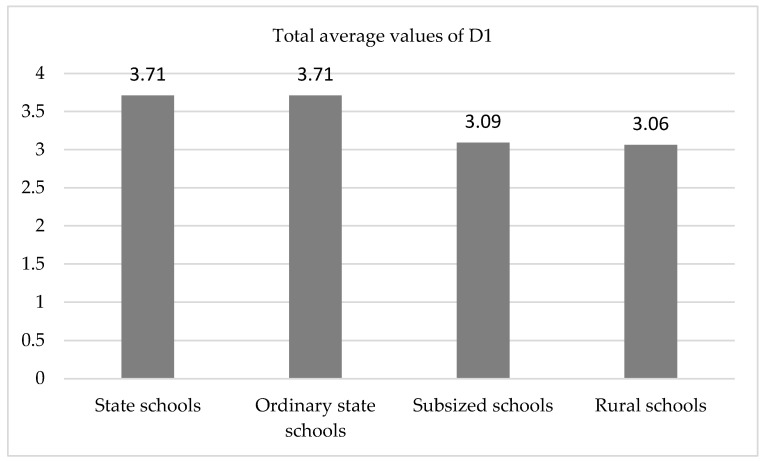
Diagram of the means. Overall mean values of D1 as a function of the type of school (*p* < 0.05).

This implies that:

- In D1, considered as a whole, a significant difference was found (*p* < 0.05), which corresponds to a high effect size (46.2%). We can, thus, conclude that there is solid evidence of statistical differences between the different types of schools. The data we have (Figure 1) indicate that the lowest means that result in this difference were observed in subsidized and RAS schools. It is in these schools where non-marked dangerous areas were observed in the spaces utilized for recess. Most of these were not limited to the play areas.
- The cause of the overall significant difference for D1 was especially found in items 1.2 (The play space for recess is well marked) (*p* < 0.01) and 1.5 (Safe use of equipment) (*p* < 0.001), where the size effects were very large (54.5% and 67.3%), with the worst result in the RAS, where a clear delimitation of the different play spaces was not found.
- Significance was almost found (*p* < 0.10) with large effect sizes (between 28.5% and 33.9%) in items 1.1 (Unsafe areas are identified and well marked) and in the second observation of item 1.3 (Safety of fixed and non-fixed recess equipment, Observation during a rainy day). The lowest results were again found in the RAS and subsidized schools.

When focusing on the transitions considered in D4, practically all the mean values were found to be between 2 and 3 points, that is, in the middle. The results of the comparison of the three observations showed that although some differences were observed in item 4.2 (Organization of transitions from recess to the classroom), they were not statistically significant (*p* > 0.05).

As for item 4.1 (Organization of transitions from the classroom to recess), a high level of significance was found (*p* < 0.01) for differences between the assessments from the first observation and the other two, with an effect size of 28.4% (therefore very high). As a result, we must state that between the three observations conducted in these 23 schools (Table 4), significant changes were found in the aspect assessed by item 4.1, with a decrease observed from the first observation with respect to the other two, which indicates that the morning transitions were more orderly and fluid in the morning recess on sunny days than in rainy days or at noon. No changes were found in the other items of this dimension among the three observations conducted.

**Figure 2:**
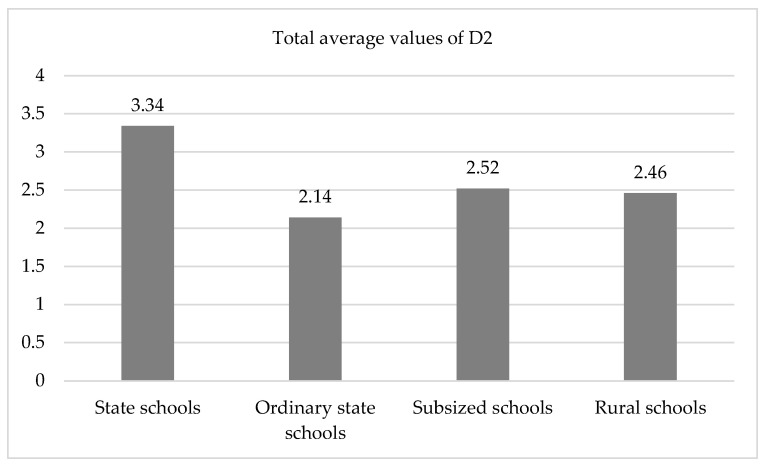
Diagram of means. Overall mean values of D4 as a function of the type of school (*p* < 0.01).

When considering the type of school in D4, we found highly significant differences in the overall score, with a very large effect size (40.2%), with the highest mean value found in the high-complexity schools and lower values in the rest, but especially in the ordinary public ones (Figure 2). Thus, in the high-complexity schools, the transitions were more orderly and fluid, which decreased the possible risks that could have occurred, as opposed to what was found in the other types of schools, especially in the ordinary ones.

In the first two observations of item 4.1 (Organization of transitions from the classroom to recess) (Table 5), highly significant values were found (*p* < 0.001 and *p* < 0.01) with very high effect sizes (61.3% and 43.9%), as shown by the very low values, especially in ordinary schools. This indicates that in these schools, little or no transitions were conducted in an orderly or fluid manner. On the third observation, we did not find a significance (*p* > 0.05), although the effect size should still be taken into account (17.1%). The differences in the transitions according to the type of school indicate how the transitions in high-complexity schools, in every observation, were organized and fluid, reducing the possible risks.

A high level of significance (*p* < 0.01) was also found in item 4.2 (Organization of transitions from recess to the classroom), with very large effect sizes (66.3% and 51.9%, respectively). This was due to the high values found for the high-complexity schools, indicating that the adult supervisors arrived on time, the students did not have periods of time without adult supervision, and the transitions were fluid and organized.

### 3.2. Student Empowerment

The same statistical test as for the previous dimensions was performed with the items from the D3: Student behaviors, with the same consideration with respect to the scale, although the value of 5 was added as a representation of the absence of problems/risks.

In the descriptive analysis of these items, we found observations distributed throughout all the values, with a predominance at both ends, although these were slightly higher on the positive end (3-4-5). This is the reason why the means were all above 3, which indicates a trend or that no problems/risks existed, or that if these were present, they were resolved.

**Figure 3:**
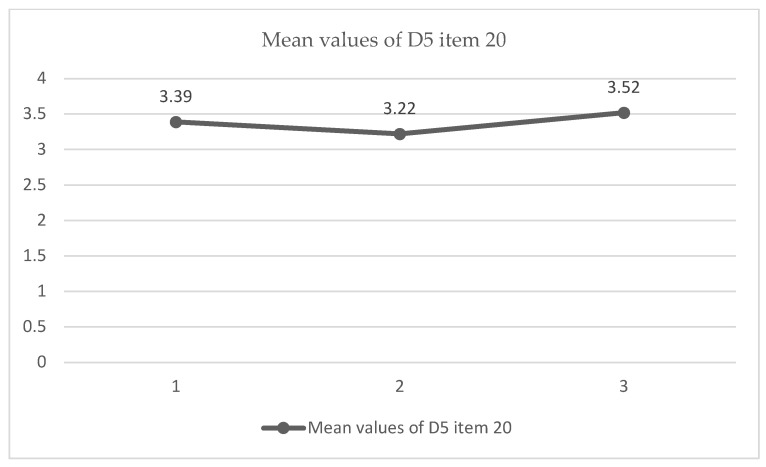
Diagram of means. Comparison of item 20 between all 3 observations (*p* < 0.05) (*n* = 23).

When comparing the three observations performed for item 3.5 (Students/resolution strategies), a slightly significant value was found (*p* < 0.05), accompanied by a moderate–high effect size (11.5%), which leads us to conclude that there were changes between observations. The data collected (Figure 3) indicate that this significance was due to the increased score in the third observation with respect to the previous two. It seems that in the noon observation, the students had to utilize strategies to resolve conflict and situations of risk without adult intervention. Students used mediation and conflict resolution strategies. Moreover, they did so autonomously, without adult help. All types of risks were assessed: physical, behavioral, and interpersonal.

**Figure 4:**
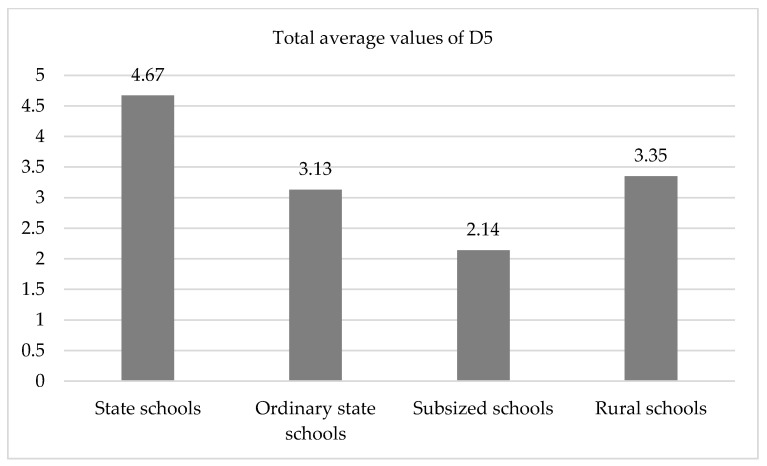
Diagram of means. Overall mean values of D3 as a function of type of school (*p* < 0.001).

The results from the comparisons of the D3 variables show:

- A high level of significance in the total D3 score (*p* < 0.001) with an effect size of 49.3% (very large), which was produced (Figure 4) by both the high value in the high-complexity schools, as well as the low value observed in the subsidized ones. Thus, in the high-complexity schools, disagreements were not found between students with respect to the guidelines, and they themselves applied strategies to resolve situations of risk without adult intervention, which did not occur in subsidized schools, where the students did not seek strategies by themselves, but instead sought adult intervention to revolve risk situations. Additionally, the students did not know the guidelines, as there were many disagreements about their definition and application. It is worth remembering that, in the GRF-OT.Cat observation scale, there are different observable patterns. It was not the specific strategies that were analyzed, but whether the conflict, in general, was solved or not. The scale, also, allows observers to analyze whether students demand the attention of supervisors, not the other way around. In this case, highly complex schools stood out because students solved conflicts autonomously. In contrast, in subsidized schools, students demanded teachers’ attention in order to resolve conflicts. In this respect, it should be noted that subsidized schools have a lower ratio of students to supervisors.
- This same trend, with significant values (*p* < 0.01) and large effects (between 4.1% and 56.6%) were found in the three measurements of item 3.5 (Students/resolution strategies). This shows that independently of the moments during the school day in which the observations were made in the high-complexity schools, the students were the ones who resolved the situations of risk without the intervention of the adults.
- As for item 3.4 (Students/disagreements about guidelines), a significance was not found (*p* > 0.05), although a large effect size was observed (37.6%), which allows us to admit that differences were present. These differences were especially due to the low score from the subsidized schools, where the students disagreed and were unaware about the guidelines.

## 4. Discussion

Some researchers [3,4,5,6,7,9,10,47] believe that school recesses are key and essential for each student’s learning. In these spaces, safety is another objective, just as in any other activity conducted in the education center. Nevertheless, the availability of norms and guidelines that rule the facilities, the resources, and their safety does not always signify that the educational activity is exempt from risks.

When making observations at three different moments in time during the school day, significant differences were detected between the three different recess periods analyzed, in favor of the morning recess. The different recess periods had specific characteristics, such as duration and the organization of the supervision. Thus, the noon recesses lasted the longest, in 2–3 h periods, and were preferably supervised by recess monitors. However, the morning recesses usually lasted 30 min, and the supervision was mainly provided by teachers. These specific peculiarities could be one of the reasons for the results mentioned above, in addition to the fact that, during the morning, supervision is exercised by more qualified personnel, i.e., teachers. It is important, then, for both teachers and noon monitors to receive training on guidelines related to security issues and risks in spaces where recess takes place [34,35].

Some researchers [39,40,42,45] believe that the transitions between different spaces were also an issue of interest, not only from the classroom to recess, but also with the entry and exit from the school. In the present study, the high-complexity schools were highlighted, as the transitions there were orderly and fluid, thus reducing the factors of risk that could be created during the entry and exit from the center. The same authors appealed to the importance of having organized transitions, without the need to make the students form a line, as long as they knew the center’s guidelines with respect to the transitions. Orderly and fluid transitions are understood as those from the classroom to the playground, from the playground to the classroom, or to the exits and entrances of the school, wherein pupils and adults are grouped together, but the passage from one space to another is made quickly, without disturbances, without interruptions, without obstacles, and without crowding.

Highly complex schools have a socio-economically disadvantaged population and have lower results in the external evaluation of competences than other schools. A priori, they are schools where there may be more conflict, and this may generate more risk situations. However, the results obtained indicate that it is where safety is most worked on and where pupils are more aware of the rules of coexistence, and where they are, thus, more proactive in their application without adult intervention. We suggest that this way of dealing with safety issues in highly complex schools can serve as a reference for practice in other schools. Observations show that students enforced the rules among themselves and did not turn to the adults to solve possible conflicts.

Another significant result was that in rural and subsidized schools, we found non-marked areas of risk, and also, the play areas were not well defined compared to the other types of schools analyzed. We can say, then, that risks existed in subsidized and rural schools, although it is prescriptive to promote the maximum level of safety possible at all times [19,23,28].

Contrary to this, and in agreement with [29,30,48,49], high-complexity schools were noteworthy, as the play areas were correctly defined, and the risk areas were well marked. The contrast between the subsidized, rural, and high-complexity schools is explained, among other reasons, by the characteristics of the students they serve. The authors of [38,46] indicated that schools which serve vulnerable populations from unfavored socio-economic contexts tended to have the highest levels of conflict. This obliges the teachers to exhaustively plan the co-habiting and use of space guidelines to ensure the reduction of possible risks that could trigger new conflicts or accidents that could ultimately affect the students.

Some authors [38,46] have highlighted that at subsidized schools, which tend to service students from medium and medium–high socio-economic levels, and at rural schools, which serve students from small towns where most residents know each other, the indices of conflict are lower. This is the reason why the teachers do not set limits in the different play areas, thus allowing, for example, the interaction between students of different ages, without it resulting in problems of safety. This is also true when an issue appears at recess, which, in these cases, is not properly pointed out.

In turn, subsidized schools lead the students to look for the figure of an adult to resolve conflicts, as evidenced by [38,47,48,50,51]. Curiously, this situation is not found here, as the students demonstrated their knowledge about the guidelines and their application, independently acting without the need for adult intervention, as some researchers described in [29,30]. The greater level of student empowerment shown at high-complexity schools makes them co-responsible and participants in school safety, as indicated by [38]. If the students are empowered at schools to be responsible for their own safety and self-care, then they could extrapolate these attitudes outside the school to independently deal with any situation of risk [50]. This means that just as authors [49,51,52,53] have indicated, working on the subjects of security at schools with the participation of the students could be something that can be transferred to their everyday realities.

The design of playground spaces in schools is an absolute necessity [35,36], as is the involvement of the different educational agents in this process. Some researchers [37] point out that there is a link between the design of the school space and its safety and risk minimization. Therefore, the planning of such spaces and their maintenance should be a key objective of schools.

Ultimately, as shown by [50,51], an essential objective of schools is to empower the students with the knowledge of the guidelines to self-protect against the possible risks. All of this signifies that teachers must make students also responsible with the writing of the guidelines, their compliance, and their evaluation.

## 5. Conclusions

This study has provided evidence about the analysis of safety and risk in school transitions and recess and the empowerment of students to manage situations of risk, which were the proposed objectives.

Schools, regardless of their type, must comply with safety standards, as set out in the regulations. However, this does not mean that there are no risks during recesses and transitions, and therefore schools must be aware of them and seek ways to minimize or solve them.

This study has shown that when students are clearly aware of recess rules and guidelines, as is the case in highly complex schools, the management of conflict situations improves. We conclude, therefore, that the work model of high-complexity public schools, in which, due to the context, risk and conflict management is worked on more consciously, could be extrapolated to other types of schools, where in principle there is no need for it. However, it is like a snake biting its own tail: it is not worked because in theory the context is less conflicting, but, precisely for this reason, in the end there are more problems than expected.

Additionally, it is necessary to favor training related to supervision during recess for teachers and noon recess monitors. Adult supervisors must be trained on safety and risk issues in education center settings.

Due to the COVID-19 pandemic, schools were subjected to a series of risks that were not expected, and education centers, remarkably, knew how to resolve these issues. They had to plan the transitions, the recesses were planned at different times, and the play areas were delimited, considering the groups, etc.

It is necessary to highlight that one of the most important mechanisms to overcome this situation was the empowerment of students, as they were aware that they had to follow the established guidelines (maintain the distance of safety, use a mask, etc.) in line with what was already occurring in high-complexity schools to improve safety. This is a clear example of the position of the present article: students must know how to self-protect.

The present pandemic has provided evidence on different safety and risk problems, and it would be interesting to know if education centers, beyond the high-complexity schools which had already developed these mechanisms, have continued to maintain the empowerment of students on safety and risk issues. Observations could be made if the space limits according to groups are still in use, if they are still planning the transitions according to different times, and if they are still promoting the empowerment of students beyond safety and self-protection issues.

The study showed limitations due to the use of a quantitative methodology. This study could be complemented with the use of qualitative tools that could complete the results.

Finally, our study, unique in our context, opens up new lines of research. For example, it could be reproduced in schools throughout Spain in order to compare and corroborate results. In addition, it would be interesting to compare the management of safety and risk situations in school transitions and breaks between the school model we have analyzed (children take long breaks after several class sessions) and other models in which they take short breaks after each class session.

## Figures and Tables

**Table 1:** Dimensions and items used form the GRF-OT scale.

Dimensions	Items
D1. Safety and structure	1.1. Unsafe areas are identified and well marked. 1.2. The play space for recess is well marked. 1.3. Safety of fixed and non-fixed recess equipment. 1.5. Safe use of equipment.
D3. Student behaviors	3.4. Students/disagreements about the guidelines. 3.5. Students/resolution strategies.
D4. Transitions	4.1. Organization of transitions from the classroom to recess. 4.2. Organization of transitions from recess to the classroom.

**Table 2:** Comparative analysis of observations. Items from D1: Safety and structure. GRF-OT.Cat. *n* = 23 schools.

Item	Mean/Median Values			Friedman Test	
	1st Obs.	2nd Obs.	3rd Obs.	Value	*p*-Sig
1.1. Unsafe areas are identified and well marked	3.65/4.00	3.48/4.00	3.65/4.00	1.00 ^NS^	0.739
1.2. The play space for recess is well marked	3.04/4.00	2.96/4.00	3.00/4.00	1.62 ^NS^	0.519
1.3. Safety of fixed and non-fixed recess equipment	3.78/4.00	3.52/4.00	3.52/4.00	8.00 *	0.025
1.5. Safe use of equipment	3.52/4.00	3.43/4.00	3.30/3.00	4.16 ^NS^	0.119

N.S. = not significant (*p* > 0.05); * = significant at 5% (*p* < 0.05); Obs.= Observation.

**Table 3:** Differences in means. Comparison of the significance of the differences in the variables from D1: Safety and structure as a function of school type. (*n* = 23).

Item	Mean/Median According to Type of School				KW Test		Effect Size R^2^
	High-Complexity	Ordinary	Subsidized	Rural Area Schools	Statistic	*p*	
Total D1	3.71/3.71	3.71/3.71	3.09/2.83	3.06/3.19	9.50 *	0.023	0.462
1.1	3.89/4.00	3.83/3.83	3.28/3.17	3.33/3.00	6.90 ^NS^	0.067	0.339
1.2	3.06/3.17	4.00/4.00	3.00/3.00	1.73/1.67	13.27 **	0.001	0.545
1.3/1st obs.	4.00/4.00	4.00/4.00	3.50/3.50	3.60/4.00	6.82 ^NS^	0.057	0.310
1.3/2nd obs.	3.50/3.50	4.00/4.00	3.17/3.00	3.40/3.00	6.41 ^NS^	0.089	0.285
1.3/3rd obs.	3.83/4.00	3.50/3.50	3.17/3.00	3.60/4.00	3.53 ^NS^	0.329	0.177
1.5	4.00/4.00	3.83/3.83	2.61/2.33	3.20/3.00	14.75 **	0.000	0.673

N.S. = not significant (*p* > 0.05); * = significant at 5% (*p* < 0.05); ** = highly significant at 1% (*p* < 0.01); Obs.= Observation.

**Table 4:** Comparative analysis of observations. Items from D4: Transitions. GRF-OT.Cat. *n* = 23 schools.

Item	Mean/Median Values			Friedman Test	
	1st Obs.	2nd Obs.	3rd Obs.	Value	*p*-Sig
4.1. Organization of transitions from the classroom to recess	3.04/3.00	2.52/3.00	2.48/2.00	13.51 **	0.001
4.2. Organization of transitions from recess to the classroom	2.74/3.00	2.30/2.00	2.39/2.00	5.14 ^NS^	0.107

N.S. = not significant (*p* > 0.05); ** = highly significant at 1% (*p* < 0.01); Obs. = Observation.

**Table 5:** Difference in means. Comparison of the difference in the variables from Dimension 2: Transitions at recess, as a function of the type of school. (*n* = 23).

Item	Mean/Median according to Type of School				KW Test		Effect Size R^2^
	High-Complexity	Ordinary	Subsidized	Rural Area Schools	Statistic	*p*	
Total D4	3.34/3.28	2.14/2.08	2.52/2.56	2.46/2.50	11.98 **	0.007	0.402
4.1/1st Obs.	3.33/3.00	2.00/2.00	3.00/3.00	4.00/4.00	15.69 **	0.000	0.613
4.1/2nd Obs.	3.17/3.00	2.00/2.00	2.00/2.00	3.00/3.00	10.61 **	0.007	0.439
4.1/3rd Obs.	3.00/3.00	2.00/2.00	2.50/2.50	2.40/2.00	2.47 ^NS^	0.516	0.171
4.2	3.61/3.67	2.33/2.00	2.44/2.67	1.33/1.33	10.17 **	0.009	0.519

N.S. = not significant at 5% (*p* > 0.050); ** = highly significant at 1% (*p* < 0.010); Obs. = Observation.

## Data Availability

The data presented in this study are available upon request from the corresponding author. The data are not available to the public due to the conditions of the project contract with the funder: DOTS University Chair (Chair for the Development of Healthy and Sustainable Organizations and Territories).
